# Editorial: Revisiting the Metastatic Cascade—Putting Myeloid Cells Into Context

**DOI:** 10.3389/fonc.2020.631278

**Published:** 2020-12-23

**Authors:** Panagiota S. Filippou, George S. Karagiannis

**Affiliations:** ^1^ School of Health & Life Sciences, Teesside University, Middlesbrough, United Kingdom; ^2^ National Horizons Centre, Teesside University, Darlington, United Kingdom; ^3^ Department of Anatomy and Structural Biology, Albert Einstein College of Medicine, Bronx, NY, United States; ^4^ Integrated Imaging Program, Albert Einstein College of Medicine, Bronx, NY, United States; ^5^ Gruss-Lipper Biophotonics Center, Albert Einstein College of Medicine, Bronx, NY, United States

**Keywords:** myeloid cell, macrophage, neutrophil, myeloid-derived suppressor cell, metastasis, platelet, monocyte

## An Overview of Myeloid Cell Populations in the Tumor Microenvironment

In the tumor microenvironment, there is a wide variety of non-tumor cells, including immune cells, which participate in reciprocal interactions with tumor cells to promote the acquisition of critical cancer hallmarks (1, 2). In hematopoiesis, immune cells of the myeloid lineage arise from the common myeloid progenitor, also known as “myeloid stem cell”. Myeloid cells represent a proponent compartment of the tumor microenvironment, and comprise both terminally-differentiated cells (such as macrophages, neutrophils, eosinophils, basophils, mast cells) and more immature or undifferentiated subsets (such as monocytes), among others (3). This Research Topic focuses on novel mechanistic insights on the intricate role of myeloid cells in cancer metastasis and highlights translational and clinical opportunities.

Myeloid cells are recruited within tumor microenvironments *via* appropriate cytokines and chemokines (4), which may also serve as signals of active inflammatory process, based on the traditional conjecture that *tumors resemble wounds that never* heal (5). Recent developments in this aspect are provided in a concise review by Pia Protti and De Monte, which examines the central regulatory role of the inflammasome in the production and secretion of proinflammatory cytokines to support cancer progression. The authors elaborate on the rationalized targeting of the inflammasome *via* pharmacological strategies to suppress tumor-promoting myeloid cells.

Myeloid cells display extreme plasticity, polarized behavior and diverse functions, which may range from purely tumor-promoting to tumor-suppressive, depending on the context (6). For example, Ihle et al. have specifically profiled and investigated the role of the Bone Morphogenetic Protein (BMP) pathway in myeloid cells. Against the backdrop of a dichotomous role for the BMP pathway in cancer progression (7), the authors conclude that conditional deletion of BMPR1a in the myeloid cell lineage blocks myeloid cell differentiation capability in various hematopoietic sites, and restricts tumor progression in a syngeneic mouse model of prostate cancer. A completely different, but also cutting-edge example of myeloid cell plasticity and functional heterogeneity in the tumor microenvironment is provided in a prominent review by Kim et al., whereby the concept of “immune cell disparity” is introduced. The authors discuss critical microanatomical differences among cancer patients of diverse racial backgrounds, with a particular emphasis on myeloid cell populations (macrophages, neutrophils, myeloid-derived suppressor cells), which account for clinically observed racial differences in metastatic outcome (8). The authors propose the development of race-specific biomarkers and therapeutic targets based on myeloid cell disparity, further promoting the vision of personalized cancer medicine.

The presence of myeloid cells in the tumor microenvironment can not only influence the onset and progression of the neoplastic disease as described, but also radically affect therapeutic responses and patient outcomes (9). In an elegant review article, Neophytou et al. have underscored the regulatory roles of myeloid cells in modulating therapeutic responses and also offered a viewpoint towards the rationalized exploitation of the myeloid microenvironment to enhance therapeutic efficacy of both current and future anticancer treatment modalities. In this regard, an in-depth investigation of myeloid cell (patho)biology is crucial for deciphering the complex cellular/molecular circuitries governing cancer progression and therapeutic responses.

## Emerging Roles of Tumor-Associated Macrophages in Cancer Metastasis

Undeniably, the most well-studied myeloid cells in the context of neoplastic disease are *macrophages*, and their predecessors, *monocytes*, which under the influence of tumor cell-secreted factors, alter their functional polarization and turn into *tumor-associated macrophages* (TAMs). Traditionally, TAM-mediated alterations in extracellular matrix composition and organization have been considered as seminal factors dictating metastatic progression (10, 11). However, the heterogeneous nature of the extracellular matrix and the transient nature of TAM activation have made the in-depth examination of TAMs in their native tumor microenvironments a rather challenging task. In this regard, Hoffman and Ponik examine biomechanical aspects of the tumor microenvironment, and further discuss certain technological innovations that can be used to circumvent the aforementioned barriers and enhance our understanding on immune cell mechano-transduction.

Recent advances on the roles of TAMs in cancer progression have unraveled an unexpectedly large repertoire of structurally and functionally distinct TAM subsets, which reside in specialized microanatomical niches within the tumor microenvironment (10, 12). In this regard, the comprehensive review article by Larionova et al. has compiled existing evidence on different TAM subpopulations in the context of cancer metastasis. *Via* thorough literature integration on common cancer types (breast, colorectal, lung, ovarian, prostate), which all frequently give life-threatening metastasis, the authors provide a perspective on TAM heterogeneity along with its translational and clinical implications. In an excellent research study, Ibrahim et al. have used single-cell RNA sequencing to thoroughly characterize and compare macrophage subset diversity between pre-invasive and locally invasive lesions in preclinical mouse models of breast cancer metastasis. This analysis not only unravels TAM heterogeneity in early progression, but also provides modern insight into early intervention strategies. In the context of metastatic disease on the other side, Coste et al. have identified a novel proangiogenic TIE2^+^ macrophage subset, functionally associated with the neo-vasculature of established lymph node metastases to regulate cancer cell re-dissemination to tertiary metastatic sites. Using state-of-the-art multiphoton intravital imaging to track down individual cancer cells in the act of dissemination, the authors propose that pharmacological targeting of TIE2^+^ macrophages can be justified as a putative anti-metastatic therapy, especially useful after surgical removal of the primary tumor.

Despite currently underrepresented in the literature, the functional heterogeneity, roles and systemic (re)programming of monocytes in cancer progression cannot be neglected and are thus explored in an elegant review by Kiss et al. The authors provide a critical update on the regulation of monopoiesis by neoplastic tissue, and offer a fertile discussion on translational and clinical opportunities arising from traditional and emerging research on monocyte biology.

## Emerging Roles of Myeloid-Derived Suppressor Cells in Cancer Metastasis

Myeloid-derived suppressor cells (MDSCs) represent a heterogeneous subset of myeloid cells with immunosuppressive properties, capable of sustaining the metastatic process (13). Trovato et al. provide a state-of-the-art update on past and recent developments, with an equal emphasis in the role of MDSCs in each fundamental step of metastatic progression. Nevertheless, one of the most puzzling questions in the field for quite some time has been the origins of MDSCs, since investigators have described multiple subsets in the past (14). A few theories and controversies on the topic are critically discussed in a review by Bergenfelz and Leandersson, who classify MDSCs in two broad categories, the granulocytic (G-MDSCs), and the monocytic (Mo-MDSCs), as defined by surface phenotype and functions. Following a slightly different paradigm of MDSC classification, two linked studies by the same research group, the first led by Saleh et al. and the second led by Nair et al., collectively investigate the mechanistic and clinical implications of different MDSC subsets in colorectal cancer (CRC) patients. Using sophisticated sorting and transcriptomic approaches, the group report on critical molecular pathways and signatures prompted by either polymorphonuclear MDSCs (PMN-MDSCs) or immature MDSCs (I-MDSCs), including associations of the resulting signatures with disease progression and metastatic outcomes in CRC patients. It is undisputable that MDSCs represent a critical component of the tumor microenvironment and play key roles in metastatic progression, but to our opinion, a consensus needs to be reached with regards to more accurate definition and taxonomy.

## Emerging Roles of Neutrophils in Cancer Metastasis

Like most other myeloid cells discussed, neutrophils are also recruited in the tumor microenvironment *via* specific neoplastic signals, to exert either tumor-promoting or tumor suppressive effects (15). In an excellent review by Kalafati et al., the authors summarize both traditional and emerging knowledge on neutrophil involvement in tumor and metastasis establishment, and further speculate on the development of neutrophil-targeting therapies. Furthermore, recent advances in neutrophil biology have underscored their implication in immunosuppression, now considered among the critical cancer hallmarks supporting both the development and the progression of cancer (15). It is yet unclear whether such neutrophils, also known as granulocytic myeloid-derived suppressor cells (g-MDSCs), also exert antimicrobial activity, or become completely repolarized and committed to the immunosuppressive tumor microenvironment. In pursuit for an answer, Aarts et al. elegantly demonstrate that granulocytic colony-stimulating factor- (G-CSF)-dependent mobilization of transfused neutrophils in chemotherapy-induced neutropenic patients can silence the g-MDSC activity, without affecting their antimicrobial activity. Overall, tumor-associated neutrophils may play pleiotropic roles in cancer progression, and future therapeutic strategies should consider targeting their tumor-promoting properties, while preserving their antimicrobial activities.

## Emerging Roles of Platelets in Cancer Metastasis

Despite that megakaryocytes and their cellular fragments, the platelets, also represent pure progeny of the myeloid lineage (16), they are often disregarded as critical constituents affecting the metastatic cascade. In this context, Lucotti and Muschel provide an exemplary review on the molecular dialogue between cancer cells and megakaryocytes/platelets in driving the metastatic phenotype. Despite that platelet coating in the bloodstream appears to be the principal mechanism that promotes cancer cell survival and facilitates the metastatic cascade, other paracrine/juxtacrine mechanisms are also crucial in either the primary tumor or the secondary metastatic site. Especially for the latter, a supportive mini review article by Gkolfinopoulos et al. provides mechanistic insights with regards to the contribution of platelets to the formation and expansion of the early micrometastatic niche, a nurturing environment that supports subsequent development of overt metastases. Both articles discuss exciting possibilities of translating these insights into the clinic, in particular through the incorporation of anti-coagulant therapy in cancer treatment.

## Conclusion

Given the detailed complexities of the myeloid cell repertoire in the tumor microenvironment, it is beyond the scope of this Article Collection to provide exhaustive information on the topic. On the contrary, here, we aim to promote a universal understanding on the involvement of key players of the myeloid lineage in the metastatic cascade. By assorting notable research and review articles, written by prominent groups in this unique field of research, we anticipate to successfully introduce the topic to new investigators, and also provide a reasonable framework to more established researchers, for bridging basic knowledge on myeloid cell biology with translational and clinical opportunities in the cancer field.

## Author Contributions

All authors contributed to the article and approved the submitted version.

## Conflict of Interest

The authors declare that the research was conducted in the absence of any commercial or financial relationships that could be construed as a potential conflict of interest.
